# Current State and Future Directions of Genetics and Genomics of Endophytic Fungi for Bioprospecting Efforts

**DOI:** 10.3389/fbioe.2021.649906

**Published:** 2021-03-15

**Authors:** Rosa Sagita, Wim J. Quax, Kristina Haslinger

**Affiliations:** Groningen Institute of Pharmacy, Chemical and Pharmaceutical Biology, University of Groningen, Groningen, Netherlands

**Keywords:** genome mining, biosynthetic gene cluster, biosynthetic pathway elucidation, culture-dependent, culture-independent, secondary metabolite discovery

## Abstract

The bioprospecting of secondary metabolites from endophytic fungi received great attention in the 1990s and 2000s, when the controversy around taxol production from *Taxus* spp. endophytes was at its height. Since then, hundreds of reports have described the isolation and characterization of putative secondary metabolites from endophytic fungi. However, only very few studies also report the genetic basis for these phenotypic observations. With low sequencing cost and fast sample turnaround, genetics- and genomics-based approaches have risen to become comprehensive approaches to study natural products from a wide-range of organisms, especially to elucidate underlying biosynthetic pathways. However, in the field of fungal endophyte biology, elucidation of biosynthetic pathways is still a major challenge. As a relatively poorly investigated group of microorganisms, even in the light of recent efforts to sequence more fungal genomes, such as the 1000 Fungal Genomes Project at the Joint Genome Institute (JGI), the basis for bioprospecting of enzymes and pathways from endophytic fungi is still rather slim. In this review we want to discuss the current approaches and tools used to associate phenotype and genotype to elucidate biosynthetic pathways of secondary metabolites in endophytic fungi through the lens of bioprospecting. This review will point out the reported successes and shortcomings, and discuss future directions in sampling, and genetics and genomics of endophytic fungi. Identifying responsible biosynthetic genes for the numerous secondary metabolites isolated from endophytic fungi opens the opportunity to explore the genetic potential of producer strains to discover novel secondary metabolites and enhance secondary metabolite production by metabolic engineering resulting in novel and more affordable medicines and food additives.

## Introduction

Endophyte is an all-encompassing term that refers to organisms which, within a certain period of their life, colonize the interior organs of their plant hosts (Box 1). Among them, endophytic fungi represent one of the largest communities with conservatively estimated at least 1 million species (Rashmi et al., 2019). More than 100 years of research indicate that most, if not all of the billions of living land plants are host for different endophytic fungi in natural ecosystems. This makes this group of microorganisms one of the largest untapped natural resources for the bioprospecting of secondary metabolites and biosynthetic enzymes (Manganyi and Ateba, 2020).

Box 1 **Definition of “endophyte.”**

The molecular mechanisms underlying endophytism are to this date uncertain and several fungal strains have been observed to be pathogenic in one host plant and neutral or mutualistic in another (Kogel et al., 2006). It has been speculated that endophytic lineages have evolved from plant pathogenic ancestors (Delaye et al., 2013), while other scientists argue for the opposite (Xu X. H. et al., 2014). The “endophytic continuum” model (Schulz and Boyle, 2005) suggests that the outcome of the plant-fungus interaction, which can range from mutualism to parasitism, depends on not only the fungal species, but also the host genetic background and the environment (Kogel et al., 2006). As pointed out by Delaye et al. (2013), fungi can easily switch at the evolutionary or ecological timescale between the symptomless endophytic life style in host tissue and the life as a necrotrophic pathogen that kills its host. Therefore, it is impossible to deduce the lifestyle of a fungus solely from a species database. It appears that currently the most commonly used indicator for endophytism is the healthy appearance of the host plant that the putative endophyte was isolated from. Clearly, stronger experimental evidence would be desirable, such as observations of the fungal behavior on stressed or wounded host plants, or during several developmental stages of the host plant from seed to senescence.

For the purpose of this review we use the term “endophyte” with the broad definition of a fungus isolated from surface sterilized plant material that does not show visual signs of disease. Of course, we rely on the accurate reporting of the scientists who performed the experiments and their appropriate use of experimental controls to avoid contamination from other sources. Hopefully in the future it will be easier to classify fungal isolates as endophytes by yet to be identified shared genomic or metabolomic traits.

Since 1981, almost 40% of FDA approved small molecule drugs were discovered or derived from natural sources (Newman and Cragg, 2020a), including medicinal plants and their endophytes. The discovery of endophytes dates far back to 1898 (Vogl, 1898), but it did not receive much attention until the past two decades, when it became evident that endophytes, especially endophytic fungi, harbor an enormous potential for secondary metabolite production with relevant bioactivities and diverse molecular structures, which are hardly mimicked by synthetic chemistry. Most notably, Stierle and Strobel (1993) pioneered the exploration of an endophytic fungus associated with *Taxus* spp. for its proposed independent synthesis of taxol (**1**). Numerous studies have since then reported on the isolation of new and previously known secondary metabolites from endophytic fungi as recently reviewed by Manganyi and Ateba (2020) and Newman and Cragg (2020b).

Endophytic fungi play a profound role for the survival and fitness of plants (Dubey et al., 2020; Gupta et al., 2020). Considering the intricate balance between overcoming host barriers and establishing a mutualistic relationship with the host, endophytes are assumed to adapt to their symbiotic microenvironments by genetic variation, including the uptake of foreign DNA. Such DNA uptake is overall rare in fungi, but has been shown to occur with microbial and plant donors (Richards et al., 2009, 2011; Armijos Jaramillo et al., 2013). This mechanism is often suggested to explain the detection of secondary metabolites that were originally identified as phytochemicals associated with the host plants (Strobel, 2003), however, experimental evidence has not been presented yet. The concept was furthermore refuted by Heinig et al. (2013) as they did not find genetic evidence for production of **1** in several endophytic fungi associated with *Taxus* spp. At the same time the authors identified several caveats in the experimental approach of studies detecting **1** from endophytic fungi and called for appropriate experimental controls in order to rule out the false-positive detection of metabolites and the carry-over of enzymes or nucleic acids from the host plant during isolation and cultivation of the fungus. The controversy regarding the biosynthesis of putative secondary metabolites by endophytic fungi has progressively increased since and has strikingly impacted the field.

While the controversy was sparked around the high-value phytochemical **1**, the same arguments of proper experimental controls and genetic evidence can be made for other secondary metabolites isolated from endophytic fungi. Most bioprospecting studies in the field focus on the isolation and characterization of bioactive compounds from one-time sampling instead of a time-course observation of the fungal culture. Often, the identification of the underlying biosynthetic pathway is not well-presented with an experimental verification to provide appropriate evidence of the proposed secondary metabolite production by endophytic fungi. Several studies reported instability of secondary metabolite production by the native host in axenic culture supposedly due to pathway silencing and inactivation, or enzyme attenuation (El-Hawary et al., 2016; Gupta et al., 2018; El-Sayed et al., 2019). In contrast to the widely claimed promise of endophytic fungi as a highly prolific secondary metabolite producer, secondary metabolite bioprospecting appears far-fetched at this point considering that their underlying biosynthetic pathways are elusive and that so many fungal strains are even unculturable under laboratory conditions (Wu B. et al., 2019).

In the current post-genomic era, these problems should be able to be resolved. With the advanced development of sequencing technologies, sequencing cost and turnaround time are dramatically reduced (Goodwin et al., 2016), making genomics and metagenomics widely accessible. Genetics- and genomics-based strategies have risen as comprehensive approaches to study natural products from a wide-range of organisms (Hover et al., 2018; Schorn et al., 2019; Walker et al., 2020). They allow the elucidation of underlying pathways for secondary metabolites isolated from organisms and facilitate the computational discovery of secondary metabolite biosynthetic pathways. From there, further exploration of the biosynthetic potential of a producer strain is made possible to activate silent pathways and to conduct rational *de novo* design of novel molecules.

**Figure 1:**
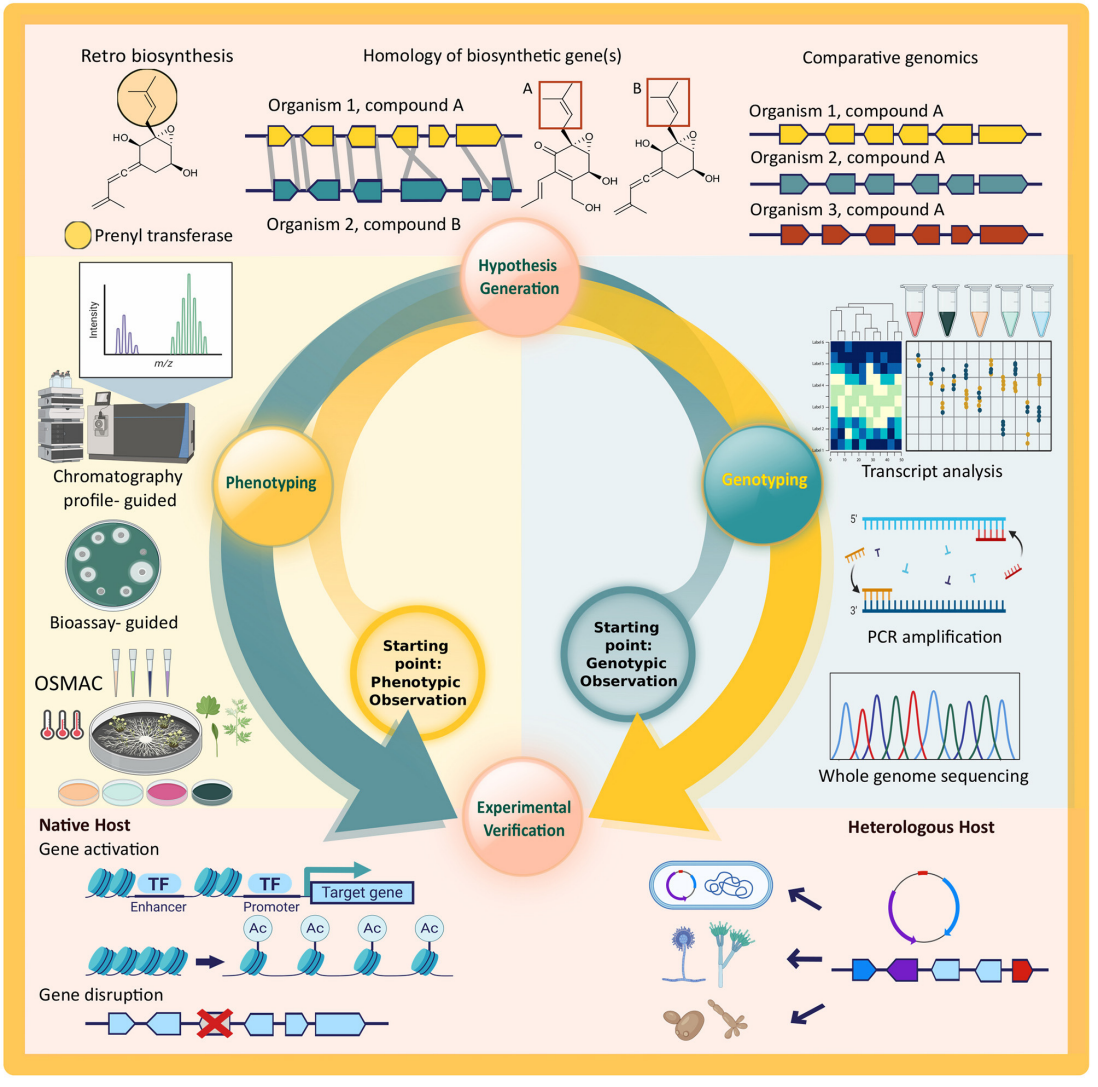
Schematic overview of secondary metabolite discovery and pathway elucidation steps discussed in this review. All steps of the cycle are required to fully connect phenotypic observations to the genotype of an organism and vice versa. Key technologies depicted for each step are: whole genome sequencing (WGS), amplicon sequencing after PCR amplification, and transcriptomics and reverse transcriptase quantitative PCR (RT-qPCR) for genotyping; retro-biosynthesis, homology searches, and comparative genomics to generate hypotheses on the link between genotype and phenotype and vice versa; chromatography- and bioassay-guided metabolite discovery and analysis, possibly coupled to differential culturing of the native host (OSMAC) for phenotyping; gene activation and deletion in the native host as well as recombinant expression of target genes in heterologous hosts for experimental verification. Created with graphical elements from BioRender.com.

However, given the scarcity of genomic information from endophytic fungi and difficulties in experimental verification of putative Biosynthetic Gene Clusters (BGCs), the majority of secondary metabolite biosynthetic pathways are still undiscovered. In this review, we will provide a brief overview of the steps needed for a successful bioprospecting study that provides both phenotypic and genotypic evidence for secondary metabolite production by endophytic fungi. As depicted in Figure 1, there are two possible starting points, but the overall steps to be taken are the same for both approaches. A study with phenotypic observation starts from the isolation and characterization of secondary metabolites (phenotyping), followed by a generation of hypothesis on the biosynthetic pathway, and genotyping of the responsible biosynthetic genes and/or cluster in the proposed pathway. On the other hand, a study might start from a genotypic observation via genome mining. This is also followed by the hypothesis generation of the biosynthetic pathway, all the way to the detection of secondary metabolite (phenotyping) in an experimental verification. Both approaches offer identification of the responsible biosynthetic genes and associating them with the expressed phenotype through experimental verification that will provide the fundamental evidence of independent secondary metabolite production by an organism.

In this review we will first provide an overview of the phenotyping efforts of endophytic fungi including the best practices and a few interesting bioactive metabolites identified with each strategy. For a larger survey of bioactive secondary metabolites reported for endophytic fungi, we would like to refer to other recent reviews by Manganyi and Ateba (2020) and Newman and Cragg (2020a). Second, genotyping strategies applied in endophytic fungi will be presented, followed by a brief overview of general strategies for hypothesis generation and experimental verification employed to establish a link between phenotype and genotype. For detailed reviews on general methods we would like to refer to the recent reviews by Hautbergue et al. (2018) and Kjærbølling et al. (2019). Third, we will highlight studies that drive the success in the bioprospecting of endophytic fungi by establishing the link between phenotype and genotype in endophytic fungi to date. Lastly, we will discuss the shortcomings and benefits of the different starting points and discuss the future opportunities in the field. Bioprospecting studies presented in this review focus on isolated fungal endophytes, and for a recent review on bioprospecting from multi-omics datasets from a wide range of organisms we would like to refer to van der Hooft et al. (2020).

## Secondary Metabolite Discovery by Metabolic Phenotyping of Endophytic Fungi

**Figure 2:**
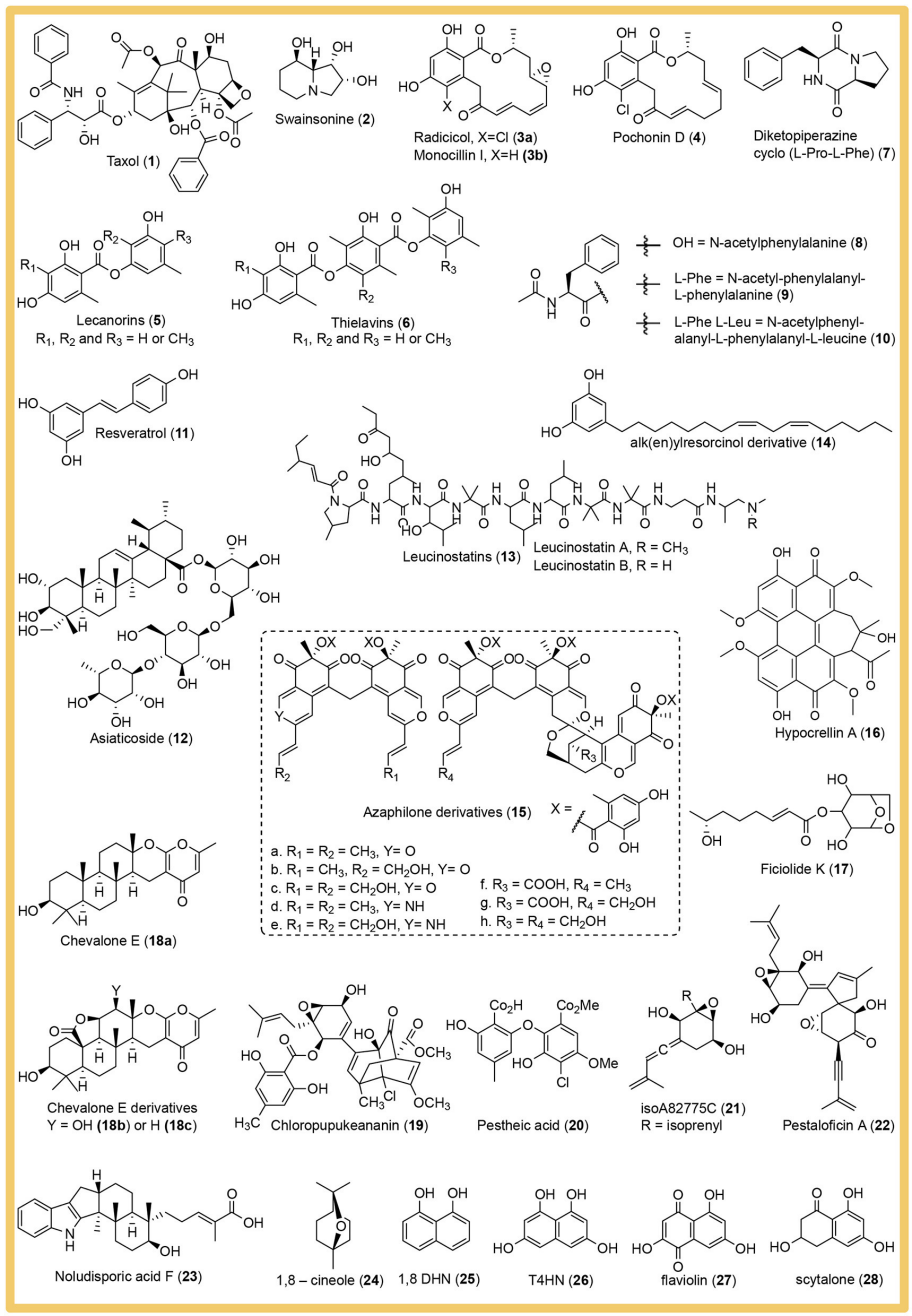
Structures of the bioactive secondary metabolites isolated from endophytic fungi and named in this review.

A large number of potentially high-value bioactive compounds with pharmaceutical importance were discovered from cultivable endophytic fungi as reviewed by Manganyi and Ateba (2020). In this review we want to focus on the main strategies employed and the best practices to prevent experimental error in phenotyping endophytic fungi. The presented examples are only a miniscule fraction of the published phenotyping studies and were selected to illustrate specific strategies. We do not intend to cast judgement on the overall quality and merit of the omitted studies. The mentioned secondary metabolites are depicted in Figure 2.

Metabolic phenotyping is often targeted toward certain chemical entities, or bioactivities (Hautbergue et al., 2018). Therefore, it is mainly based on (1) the detection of target compounds in culture extracts by chromatographic methods, either gas or liquid chromatography (GC or LC, respectively) coupled to mass spectrometry (MS) and nuclear magnetic resonance (NMR) spectroscopy (chromatography profile-guided experiment), or (2) the observation of target biological effects by a bioassay-guided experiment. An example for the first approach is a study targeting the detection of swainsonine (**2**), a cytotoxic fungal alkaloid, leading to the discovery of an independent producer of **2**, an endophytic fungus from *Astragalus mollissimus* (Braun et al., 2003). Meanwhile as an example for the bioassay-guided phenotyping approach, Turbyville et al. (2006) successfully discovered radicicol (**3a**) and monocillin I (**3b**) as Hsp90 inhibitors with potential anticancer activity in fractionated extracts of the endophytic fungus *Chaetomium chiversii* and *Paraphaeosphaeria quadriseptata*.

Instead of targeting compounds with known chemical structures or bioactivity, other studies focus on seeking “hidden gems” in metabolomic profiles of organisms to search for novel compounds, which is often employed in the study of endophytic fungi. Considering that the nature of many pathways in fungi is silent or attenuated under unfavorable experimental conditions (Keller, 2019), the qualitative and quantitative profiles of metabolites in organisms exposed to different growth conditions provide a holistic overview of their biochemical status and allows for an exploitation of their natural biogenetic capability. This approach is known under the acronym OSMAC, **O**ne **S**train-**M**any **C**ompounds (Bode et al., 2002), and has led to several interesting secondary metabolite discoveries in a wide range of organisms, including endophytic fungi (Pan et al., 2019). The discovery is mainly guided by (1) uncharacterized features in the analytical profile of fungal extracts, (2) bioassays with fungal extracts, or (3) a combination of both. Using the first strategy, novel lecanorin (**5**) and thielavin (**6**) polyketides were discovered from the extract of a guava endophytic fungus *Setophoma* sp. (De Medeiros et al., 2015). The authors studied the chromatographic profile (absorbance and MS) and discovered unknown features by comparing several different growth media. The compounds were isolated and characterized based on their absorbance, NMR, and MS spectroscopic properties for structure elucidation. The advantage of this approach is that the risk of rediscovery is decreased, and new scaffolds and molecules can be identified efficiently. However, since this approach is guided by only physicochemical traits of the compound, there is no guarantee for identifying relevant bioactivity. Therefore, other studies rely more to the bioassay-guided approach with consecutive fractionations of the fungal extract to the smallest fraction containing a single active substance with the target bioactivity. Based on this strategy, a diketopiperazine cyclo (L-Pro-L-Phe) (**7**), with antibacterial activity against *Salmonella enterica* was discovered for the first time in the endophytic fungus *Paraphaeosphaeria sporulosa* (Carrieri et al., 2020). To avoid rediscovery of known compounds, a combination of chromatography profile- and bioassay-guided isolation is preferred. With the combination strategy, Tawfike et al. (2018) investigated the metabolic profile of the endophyte *Culvularia* sp. isolated from the leaves of *Terminalia laxiflora*. First, they performed comparative metabolite profiling using high resolution LC-MS to assess the chemical diversity from different culture conditions and proceeded with interesting metabolites to the bioactivity assay on NF-κB and the myelogenous leukemia cell line K562. From there, mass spectral data of extracts with the desired bioactivity were analyzed by multivariate analysis using principal component analysis to identify the presence of exclusive metabolites. Those metabolites were dereplicated using the Dictionary of Natural Product database to prevent rediscovery and further isolated to elucidate their structures. This led the authors to discover N-acetylphenylalanine (**8**), N-acetyl-phenylalanyl-L-phenylalanine (**9**), and N-acetylphenylalanyl-L-phenylalanyl-L-leucine (**10**), which are predicted to be responsible for the observed bioactivities (Tawfike et al., 2018).

Overall, comparative metabolic profiling provides a more comprehensive and efficient approach to discover bioactive novel secondary metabolites in a high-throughput manner compared to targeting a single secondary metabolite.

### The Metabolic Phenotyping of Endophytic Fungi Is at High Risk for Errors

Many endophytic fungi were seen to be difficult to grow in axenic condition and/or to lose the production capability for secondary metabolites over repeat passages. Several factors might explain this phenomenon, e.g., the attenuation of biosynthetic gene expression, and/or the lack of pathway precursors or critical transcription factors as stimuli for secondary metabolite production during subculture (Li et al., 1998; Kusari et al., 2009a,b; Shweta et al., 2010; Vasanthakumari et al., 2015; El-Sayed et al., 2019). Due to this problem, numerous studies keep the passaging during strain isolation and cultivation to a minimum. As pointed out by Heinig et al., this may lead to carry-over of other microorganisms, plant metabolites, or enzymes, which leads to a false positive observation of secondary metabolites. It is therefore crucial to include the right experimental controls (Table 1). This could be as simple as repeat metabolite extractions to observe the build-up of metabolites over time, or the use of negative control cultures with the addition of fungicides. According to Heinig et al., the major culprit for carry-over of **1** from the host plant is its accumulation in the endophytic cell wall due to its physicochemical characteristics. To rule out the possibility of carry-over, a study by Shi et al. (2012) showed a time-course experiment on resveratrol (**11**) production by *Alternaria* sp. MG1, which showed accumulation of **11** starting at day 1 and coinciding with an increase in cell dry weight. They also showed the modulation of **11** production during optimization of the growth condition that supports the finding of independent production of **11** by the endophytic fungus. Similar observations were made by Gupta et al., who followed asiaticoside (**12**) production in an endophytic fungus associated with *Centella asiatica* over time across multiple passages. Although the secondary metabolite production was lost after eight passages, the build-up of **12** within each generation provided strong evidence for the independent production of **12** by this fungus (Gupta et al., 2018).

**Table 1:** Possible sources of error when working with endophytic fungi and commonly used or suggested preventive measures.

**No**	**Experimental step**	**Risk of errors**	**Preventive measures**
1	Isolation of fungus	• Sampling from diseased plants • Improper surface sterilization • Contamination with fungi from laboratory	• Properly document sampling site and deposit voucher specimen. • Include the solution of the “last wash” in all following experimental steps (PCR, cultivation on liquid and solid media). • Use appropriate aseptic techniques and regular controls for spore contamination of workspaces.
2	Secondary metabolite measurement from cultivated endophytic fungi	False positive detection caused by the carry-over of plant metabolites or enzymes	• Perform time course experiment to observe the secondary metabolite titer increase with biomass formation; possibly observed over several passages. • Include a negative control in the fermentation with added fungicides to detect any carry-over of plant secondary metabolites or enzymes that may contribute to the formation of the secondary metabolite. • Modulate secondary metabolite production under different culture conditions (should only be considered as hard evidence when combined with other controls, see above).
3	PCR amplification of putative biosynthetic genes	Contamination of extracted DNA with plant DNA, or the DNA of other microorganisms	• Include water controls during DNA extraction and PCR to control for reagent contamination • Include PCR reactions targeting plant housekeeping genes or taxonomic markers to check for plant DNA contamination • Use quantitative PCR against the gene of interest and a fungal housekeeping gene to compare copy numbers. • Generate vector-based genome libraries of the fungus to eliminate DNA fragments of low abundance

Despite the considerable risks of error, phenotypic observation has led to the successful discovery of numerous natural products with biological activities for almost a century since the discovery of penicillin (Aldridge et al., 1999). Alongside with its long-standing history, the analytical tools in chromatography and spectrometry (e.g., high resolution MS), data analysis (e.g., principal component analysis), and experimental strategies (e.g., OSMAC, dereplication) for metabolic phenotyping have emerged to facilitate the laborious isolation work and overcome the redundant analysis of metabolomic studies from natural sources (Covington et al., 2017). However, given the many experimental challenges associated with studying metabolites of endophytic fungi, there are a large number of studies that unfortunately lack the experimental controls and analytical depth to form reliable grounds for rational and efficient bioprospecting in endophytic fungi. But even in the carefully conducted studies reviewed in this section, often the elucidation of the underlying pathways has not been possible. In the next section we will review the opposite strategy for secondary metabolite discovery beginning with a genotypic, rather than a phenotypic observation.

## Secondary Metabolite Discovery in Endophytic Fungi Starting From Genotypic Observations

A genotypic observation can also be the starting point for a secondary metabolite discovery project. This observation can either be the presence of signature genes identified by (1) polymerase chain reaction (PCR) amplification, (2) the co-regulation of a set of genes observed on the transcript level, or (3) the computational identification of interesting BGCs in whole genome sequencing (WGS) data. In the first strategy, degenerate PCR primers are designed based on the sequences of known biosynthetic genes in order to screen isolated genomic or environmental DNA for the presence of a certain gene, and obtain its sequence. Depending on the design of the primer binding site, the screening can be highly targeted toward a gene encoding a specialized enzyme, e.g., taxadiene synthase (Staniek et al., 2009; Heinig et al., 2013), or allow for a rather untargeted detection of genes encoding an enzyme family, e.g., polyketide synthases (PKS) (Wang et al., 2008). In the case of endophytic fungi, Heinig et al. clearly demonstrated the risk for contamination with host plant DNA and false-positive detection of DNA fragments. Appropriate controls are needed to rule out these problems. One of them is to use PCR primers targeting plant housekeeping genes or taxonomic markers to check for plant DNA contamination. Another option is to perform quantitative PCR of the gene of interest and compare it to an internal control, such as single-copy housekeeping genes of the endophytic fungus. A different approach for reducing DNA contamination in the PCR amplification strategy is to generate vector-based genome libraries of the fungus, as done for *C. chiversii* using the *E. coli* F-plasmid for a fosmid library (Wang et al., 2008). Lambda phage libraries were constructed from genomic DNA from three *Taxus* spp. endophytic fungi (Heinig et al., 2013). Since the construction of these libraries is a stochastic process, DNA fragments of low abundance, such as contaminations from plant parts or environmental DNA, are more likely to be lost.

The second genotyping strategy uses transcript analysis by reverse transcription quantitative PCR (RT-qPCR) or RNA sequencing technology (RNAseq) in order to identify genes that are transcriptionally co-regulated. As an example, RNAseq of *Alternaria* sp. MG1 was done to identify putative genes involved in the proposed biosynthetic pathway of **11** (Che et al., 2016). An RNAseq library was constructed, sequenced by Illumina technology, assembled to unigenes, annotated and analyzed for gene and pathway expression. Several candidates for pathway genes were identified, although no experimental verification was presented. This strategy is particularly useful, when combined with experimental strategies to modulate gene expression, such as deletion or overexpression of a regulatory gene, or an OSMAC strategy. As an example, different culture conditions and qRT-PCR analysis were used to determine the BGC boundaries in the elucidation of the leucinostatin (**13**) biosynthetic pathway in *Purpureocillium lilacinum* (Wang et al., 2016).

The last genotyping strategy is WGS, which allows for the computational search of biosynthetic genes and gene clusters (genome mining). There are two principal strategies of *in silico* genome mining (Weber and Kim, 2016). The rule-based strategy is used to identify gene clusters based on the presence of scaffolding enzymes, such as PKSs, non-ribosomal peptide synthetases (NRPS) and terpene synthases (TS), or signatures of ribosomally synthesized and translationally modified peptides (RiPPs). This search for genes with high sequence similarity to reference genes can be done by an automated BGC finder, e.g., antiSMASH (Medema et al., 2011), or manually with the basic local alignment search tool (BLAST) (Altschul et al., 1990). A combination of BLAST, multiple sequence alignments and homology modeling is often used to manually inspect and curate the results of antiSMASH. For example in the study of 5-alk(en)ylresorcinol biosynthesis, the authors searched the genome of the endophytic fungus *Shiraia* sp Slf14 for putative type III PKS genes (Yan et al., 2018). The authors identified one gene encoding for SsARS and confirmed its PKS activity by heterologous expression in *E. coli* and yeast, followed by metabolite analysis. The second principal strategy uses a rule-independent, machine learning-based approach for automated phylogenomic analyses and/or prediction of transcriptional co-regulation of genes. It is important to note that these models are only as good as the accuracy and depth of the genomic and biochemical data used for training (Weber and Kim, 2016). When this review was written, the use of the latter approach has not been reported in endophytic fungal research. Please refer to an extensive review by Chavali and Rhee (2018) on tools and platforms for computational mining and to van der Lee and Medema (2016) for detailed computational techniques in genome-based natural product discovery in fungi.

Genome mining can be carried out under various goals, e.g., pathway elucidation of a specific secondary metabolite, homolog search for pathway engineering purposes, discovery of novel bioactive secondary metabolites, and many more (Ziemert et al., 2016). Genome mining allows the exploration of the entire genome and thus enables us to discover novel secondary metabolites. Filamentous fungi are prolific producers of bioactive secondary metabolite, yet most of them are merely expressed under experimental conditions (Keller, 2019), which prohibits their discovery through metabolic phenotyping. This is illustrated by a study in *Pestalotopsis fici* reporting only 10 out 74 gene clusters to be active under axenic conditions (Wang et al., 2015). In another study, 43 BGCs were found computationally in *Penicillium dangeardii*, but culture extracts were dominated by rubratoxins. A metabolic shunting strategy by deleting the key gene for rubratoxins biosynthesis was applied and led to the activation of cryptic BGC encoding several novel monomeric, dimeric and trimeric azaphilones (some are depicted in Figure 2, **15**) (Wei et al., 2020). Considering the vast amount of silent BGCs in endophytic fungi, genome mining can be seen as a high-throughput strategy for “treasure hunting” of novel secondary metabolites. Furthermore, it will significantly broaden the possibility of synthetic biology efforts, allowing for refactoring and *de novo* engineering of BGCs, and facilitating the systematization of BGCs to engineer novel biosynthetic pathways. However, in order to achieve these high goals, it is of utmost importance to connect the metabolomic/phenotypic with the computational/genotypic observations, regardless of the starting point of the study. In the next section we will review the steps to complete the circle.

## Connecting Genotype and Phenotype to Facilitate Bioprospecting

Regardless whether a study started from phenotypic or genotypic observation, a crucial part in conducting successful bioprospecting studies is to connect the two with experimental evidence. Initially, a hypothesis needs to be generated on the link between the observed secondary metabolite (phenotype) and the responsible biosynthetic genes or clusters (genotype). Next, an experimental investigation is required to verify the proposed hypothesis.

### Hypothesis Generation

The three main strategies for hypothesis generation are (1) retro biosynthesis, (2) homology of biosynthetic gene(s), and (3) comparative genomics, as recently reviewed (Kjærbølling et al., 2019). In brief, the retro biosynthesis approach is based on the prediction of enzymes involved in the biosynthesis of a certain (class of) secondary metabolite, as seen in the study of Hypocrellin A biosynthesis (**16**), where four enzymatic steps were predicted based on the known biosynthetic route of a structurally similar compound (Zhao et al., 2016). Retro-biosynthesis utilizes general knowledge on biosynthetic enzymes, e.g., substrate scope, reaction mechanisms, conserved domain architectures, etc. and is often combined with the other two techniques to predict a full BGC. Since no prior knowledge from related species is required, this is a commonly used strategy to propose the biosynthetic pathway of a compound with known structure, as seen in Table 2. The second strategy, homology search of gene(s), is based on the fact that enzymes of similar function and substrate scope usually share a certain degree of sequence similarity and structural similarity. Therefore, target enzymes can be identified by searching for homologs of genes of known function from other organisms, as seen in the study of the PKS encoding-gene, *pfmaE*, in the *pfma* cluster of *P. fici* (Zhang et al., 2017). Structural 3D models can be generated based on structural information from homologs, and substrate specificities can be investigated by *in silico* docking, as done on *hyp3* from *Hypoxylon* sp. (Shaw et al., 2015). The third strategy focuses on a set of genomes, which are subjected to comparative analysis to find shared BGCs across species, or presence and absence of BGCs in secondary metabolite producer and non-producer strains, respectively. This strategy led Cook et al. (2017) to successfully link secondary metabolite **2** to its *SWN* BGC across the producers. Which strategy to use critically depends on the starting point and the initial knowledge of a study. A combination of several strategies is most often needed to propose a reasonable hypothesis with a strong logic.

**Table 2:** Successful studies in linking phenotype and genotype of the related bioactive secondary metabolites from plant endophytic fungi supported by experimental verifications.

**No**	**Endophytic fungus**	**Host plant**	**Gene or cluster**	**Secondary metabolite**	**Strategies**				**References**
					**Hypothesis generation**	**Phenotyping**	**Genotyping**	**Experimental verification**	
**Starting point of study: phenotypic observation**								
1	*Pestalotiopsis fici* CGMCC3.15140	*Camilia sinensis*	*pta* and *iac* cluster	Pestheic acid and iso-A82775C (precursors of chloropupu-keananin)	Retro biosynthesis, BGC homology	Detection of target compound by LC-MS and bioassay-guided strategy in native host	Transcript analysis and WGS	Gene knock-out and rescue by complementation in native host; heterologous expression in *E. coli*.	Liu et al., 2008, 2009; Xu X. et al., 2014; Wang et al., 2015; Pan et al., 2018
2	*Ipomoea carnea* endophyte	*Ipomoea carnea*	*SWN* cluster	Swainsonine	Comparative genomics, retro biosynthesis, BGC homology	Detection of target compound by LC-MS in native host	WGS	Gene knock-out and rescue by complementation in native host.	Braun et al., 2003; Pryor et al., 2009; Oldrup et al., 2010; Santos et al., 2011; Cook et al., 2014, 2017; Lu et al., 2016; Noor et al., 2020
	*Alternaria sect. Undifilum*	*Astragalus mollissimus, Astragalus lentiginosus*							
3	*Shiraia* sp. Slf14	*Huperzia serrata*	Putative perylenequinones cluster	Perylenequinones, especially Hypocrellin A	Retro biosynthesis, BGC homology	Detection of target compounds by HPLC in native host	WGS and transcript analysis	Gene activation by elicitor overexpression combined with transcriptomic analysis in native host.	Yang et al., 2009; Yang H. et al., 2014; Zhao et al., 2016; Liu et al., 2018, 2020; Li et al., 2019
4	*Chaetomium chiversii*	Mormon tea *Ephedra fasciculata*	*nrPKS, hrPKS, rdc5, rdc1* and *radR* genes	Radicicol and Monocillin I	Retro biosynthesis, BGC homology	Bioassay-guided discovery in native host	PCR amplification	Gene knock-out in native host and targeted inactivation of a putative cluster specific regulator.	Turbyville et al., 2006; Wang et al., 2008
5	*Purpureocillium lilacinum*	ND	*lcs* cluster	Leucinostatin A and B	Retro biosynthesis, BGC homology	Detection of target compound by LC-MS in native host	WGS	Gene knock-out by homologous recombination and overexpression of cluster specific regulator in native host.	Fukushima et al., 1983; Kawada et al., 2010; Wang et al., 2016
6	*Hypoxylon pulicicidum* strain MF5954	*Bontia daphnoides*	*NOD* cluster	Nodulisporic acid F	Retro biosynthesis, BGC homology	Detection of target compounds by HPLC in native host	WGS	Heterologous expression of BGC in *Penicillium paxillin*.	Nicholson et al., 2018; Van De Bittner et al., 2018
7	*Hypoxylon* sp. E7406B	ND	*hyp3* gene	1,8-cineole	Biosynthetic gene homology	Detection of target compounds by GC-MS in native host	WGS	Heterologous expression in *E. coli*, site saturation mutagenesis and substrate scope analysis.	Shaw et al., 2015
**Starting point of study: genotypic observation**								
1	*Aspergillus versicolor* 0312 and *Aspergillus felis* 0260	*Paris polyphylla* var. *yunnanensis*	*cle* and *sre* clusters	Chevalone E and its derivatives	Retro biosynthesis, BGC homology	Untargeted chromatography-guided isolation from heterologous host	WGS	Heterologous expression of BGC *Aspergillus oryzae* with substrate feeding experiments.	Wang et al., 2019
2	*Pestalotiopsis fici* CGMCC3.15140	*Camilia sinensis*	*pfma* cluster, *rsdA* gene	DHN melanin	BGC homology	Targeted chromatography-guided isolation from heterologous and native host	WGS	Gene deletion (BGC genes and global regulators) in native host; pathway reconstitution and gene deletion in heterologous host *A. nidulans*, secondary metabolite production rescue by complementation.	Zhang et al., 2017, 2019; Zhou S. et al., 2019; Eisenman et al., 2020
3	*Shiraia* sp. Slf14	*Huperzia serrata*	*ssars* gene	alk(en)yl-resorcinol polyketides	Biosynthetic gene homology	Targeted chromatography-guided isolation from heterologous host	WGS	Heterologous expression of *ssars* gene in *E. coli*; substrate feeding experiments in *E. coli*.	Yan et al., 2018

*ND, no data*.

Since the number of phenotypic studies on endophytic fungi, which at least proposed underlying biosynthetic pathways for the observed secondary metabolites, is so vast and were recently reviewed by Dubey et al. (2020), we will not give more specific examples here. In section Successful Elucidation of Biosynthetic Pathways by Linking Phenotype and Genotype of Endophytic Fungi of this review, we provide a deeper analysis of the studies that encompass the entire pathway elucidation process and successfully connect the secondary metabolite discovery and BGC identification.

### Experimental Verification

Depending on the starting point of the study, the experimental verification will require the same sets of experiments as described in sections Secondary Metabolite Discovery by Metabolic Phenotyping of Endophytic Fungi and Secondary Metabolite Discovery in Endophytic Fungi Starting From Genotypic Observations. For a study starting from the phenotypic observation, genotyping needs to be performed with targeted or untargeted sequencing strategies, while for studies starting with an *in silico* analysis and prediction of BGCs, targeted or untargeted metabolomics need to be performed to detect the pathway products and intermediates in the native host or a recombinant host (Figure 1). Individual gene functions need to be verified by targeted overexpression or gene disruption followed by metabolite analysis. Detailed techniques and methods for this step in the workflow were extensively reviewed recently (Hautbergue et al., 2018; Kjærbølling et al., 2019).

#### Native Host Strategy

The native host strategy is used when a target microorganism is cultivable and amenable to genetic modification. The major strategies are gene deletions and gene/gene cluster activation. Gene disruption, or knock-out by homologous recombination coupled to metabolite analysis is by far the most common method for dissecting the function of genes in BGCs, since the gene disruption should lead to the absence of the target secondary metabolite and/or accumulation of pathway intermediates (Kjærbølling et al., 2019). This strategy has been successfully applied in several studies on endophytic fungi starting from phenotypic observation (see section Studies Starting From Phenotypic Observation), e.g., the elucidation of the pathway of **2** in *Metarrhizium robertsii* (Cook et al., 2017). In studies starting from genotypic observation, this approach is more challenging, since BGCs are often silent, however, some positive examples are summarized in section Studies Starting From Genotypic Observation. A general challenge in this approach is that the common strategy to delete gene(s) by homologous recombination is inefficient or even impossible often due to repeat regions (Zhang et al., 2019). This has been overcome with the recent innovation of CRISPR/cas9 (Clustered Regularly Interspaced Short Palindromic Repeats/cas9) technology in filamentous fungi, which improves recombination efficiency and allows for targeted and multiplexed gene editing without or with fewer selectable markers as reviewed by Song et al. (2019). For example, even in the presence of “TG” repeats in the promoter region that hampered conventional gene deletion by homologous recombination, CRISPR/cas9 gene editing was performed to disrupt the *pfmaF* gene in the endophytic fungus *P. fici* and thereby elucidate its role as a global regulator (Zhang et al., 2019).

Gene activation is another strategy in the native host, since many BGCs of endophytic fungi become silent under artificial experimental conditions. Physicochemical triggers and the use of interspecies crosstalk are reported to successfully activate silent genes in many microbes (Pan et al., 2019). However, the specific requirements to induce expression from such gene clusters are not well-understood, since it is not possible to predict the complex regulatory circuits involved in an endophytic fungal biosynthetic pathway. Thus, other global activation strategies, such as epigenetic re-modeling, are promising avenues toward exploring silent gene clusters. Small molecule inhibitors of DNA methylating and histone acetylating enzymes (Toghueo et al., 2020), and targeted knock-out of the corresponding genes, e.g., the *hdaA* gene (Yang X. L. et al., 2014; Mao et al., 2015; Bai et al., 2018; Ding et al., 2020) can be employed to stimulate secondary metabolite production. This is reported in the study of the endophytic fungus *P. fici*, where Ficiolide K (**17**) was found along with 14 new polyketides upon knock-out of *hdaA* (Wu et al., 2016). Another global approach was performed by Zheng et al. (2017) to discover five novel Pestaloficins, including Pestaloficin A (**22**), by deleting the *PfcsnE* gene. Other chromatin remodeling strategies remain to be explored in fungi and promise an even wider application of this gene cluster activation strategy (Collemare and Seidl, 2019). Overall, the global gene activation strategy is mainly used to discover novel compounds, but it could also be used to pinpoint specific BGCs by studying the effect of the manipulation on transcript level in endophytic fungi. As reported for other cultivable organisms (Kang et al., 2019), we see great potential for connecting phenotype and genotype with the combination of metabolomics and transcriptomics following global gene activation in endophytic fungi.

In contrast to the global gene activation approach, targeted overexpression or knock-out of specific, positive or negative transcriptional regulators, respectively, provides a precise tool to study a certain set of co-regulated genes. This method is widely applicable, as up to 50% of fungal BGCs contain putative cluster-specific regulators (Keller, 2019), and it requires less genetic manipulation compared to promoter replacement strategies targeting individual genes (Scherlach and Hertweck, 2009). As an example, knock-out of the *RadR* gene encoding the cluster-specific positive regulator was performed to characterize functional genes in the BGC of **3** in *C. chiversii* (Wang et al., 2008). In another study, Wei et al. (2020) presented experimental evidence for the putative azaphilone BGC by showing that deletion and overexpression of the cluster-specific transcription factor *danS* in the endophytic fungus *Penicillium dangeardii* lead to changes in the production levels of **15**.

Overall, the native host strategy has been applied successfully many times to investigate the gene function in endophytic fungi. However, native host strategies cannot be used for unculturable organisms or isolates that are not amenable to classic genetic modification. In section Future Directions for Successful Secondary Metabolite Bioprospecting From Endophytic Fungi, we will review some technology advances that might help to overcome this hurdle.

#### Heterologous Host Strategy

Synthetic biology offers vast opportunity to investigate the function of BGCs, even cryptic ones or those identified in metagenomic assemblies, in a heterologous host. The possibility to generate long synthetic DNA fragments (Eisenstein, 2020) and advanced DNA assembly strategies (Bartley et al., 2020) allow for synthesis of entire clusters including *de novo* design of BGCs with host promoters and regulatory elements for better heterologous expression. Selection of a host and the design of DNA constructs suitable for the host (presence/absence of introns, codon usage, choice of vectors, and burden of foreign DNA) are the most important success limiting factors in heterologous expression, but they can be overcome with new design tools. Recent advances in heterologous expression systems for fungal BGCs were recently reviewed by Qiao et al. (2019) and Lin et al. (2020).

The most commonly used bacterial hosts for BGC reconstitution are *Escherichia coli, Streptomyces*, or *Bacillus subtilis*, and the most popular eukaryotic hosts are *Saccharomyces cerevisiae* and filamentous fungi, such as *Penicillium* and *Aspergillus*. Although there are some impressive examples of complex plant (Nakagawa et al., 2016; Chen et al., 2018; Pramastya et al., 2021) and fungal (Matthes et al., 2012; Zobel et al., 2015) pathways expressed in bacteria, we have only found studies where individual, cytosolic enzymes from endophytic fungi were expressed in bacterial hosts (Shaw et al., 2015; Pan et al., 2018; Yan et al., 2018). For reconstitution of multiple genes, fungal hosts appear to be preferred (Zhang et al., 2017; Van De Bittner et al., 2018; Wang et al., 2019). This can most likely be attributed to the challenge of intron prediction, the large size of fungal BGCs, and the lack of post-translational modifications and compartmentalization/membrane trafficking in prokaryotes. Fungi are also more likely to provide the required supporting enzymes and metabolic precursors, allowing exploration of the cloned genes or clusters even with limited information on upstream and downstream pathway modules. The main difficulty is that most fungi themselves dispose of a myriad of BGCs, which may result in crosstalk with the heterologous pathway. This was observed by Xie et al. (2011) where the recombinant biosynthetic genes of the fungal endophyte triggered the inactivation of a negative regulator in the host *Fusarium*, leading to the production of the mycotoxin fusaric acid instead of the target mycoepoxydiene. In order to eliminate cross-talk between the native and the recombinant pathway, Zhang et al. (2017) chose to integrate their heterologous genes into the locus of the native pathway genes of the host and confirmed the success of their strategy on transcript level.

Besides crosstalk, the often-complex metabolite profile of filamentous fungi as prolific secondary metabolite producers themselves may obscure the products of the heterologous pathway. The recent engineering of platform strains with low secondary metabolite background, e.g., the *Penicillium rubens* 4xKO (Pohl et al., 2020) and *A. nidulans* LO8030 (Chiang et al., 2016), will help overcome this hurdle. Wang et al. (2019) used an *A. oryzae* mutant with low secondary metabolite background, which enabled them to elucidate the biosynthetic pathway of Chevalone E (**18a**) and its analogs (**18b** and **18c**) by investigating two heterologous gene clusters from the endophytic fungi *A. versicolor* 0312 and *A. felis* 0260. This study also highlights the unique capability of fungal hosts to accept large foreign DNA constructs, here divided over multiple plasmids. Other recently developed strategies, e.g., the HEx platform for *Saccharomyces cerevisiae* (Harvey et al., 2018) and the construction and delivery of fungal artificial chromosomes (Bok et al., 2015; Clevenger et al., 2017) to fungal hosts are major break-throughs in the field and will facilitate the study of BGCs from endophytic fungi in heterologous hosts.

## Successful Elucidation of Biosynthetic Pathways by Linking Phenotype and Genotype of Endophytic Fungi

Despite the previously described challenges in phenotyping and genotyping efforts, several studies were reported to successfully link secondary metabolite phenotype and its genotype supported by meticulous experimental verification, either with phenotypic observation or genotypic observation as the starting point of the study. Here we review these studies as cornerstones of future bioprospecting efforts of endophytic fungi (Table 2).

### Studies Starting From Phenotypic Observation

**Figure 3:**
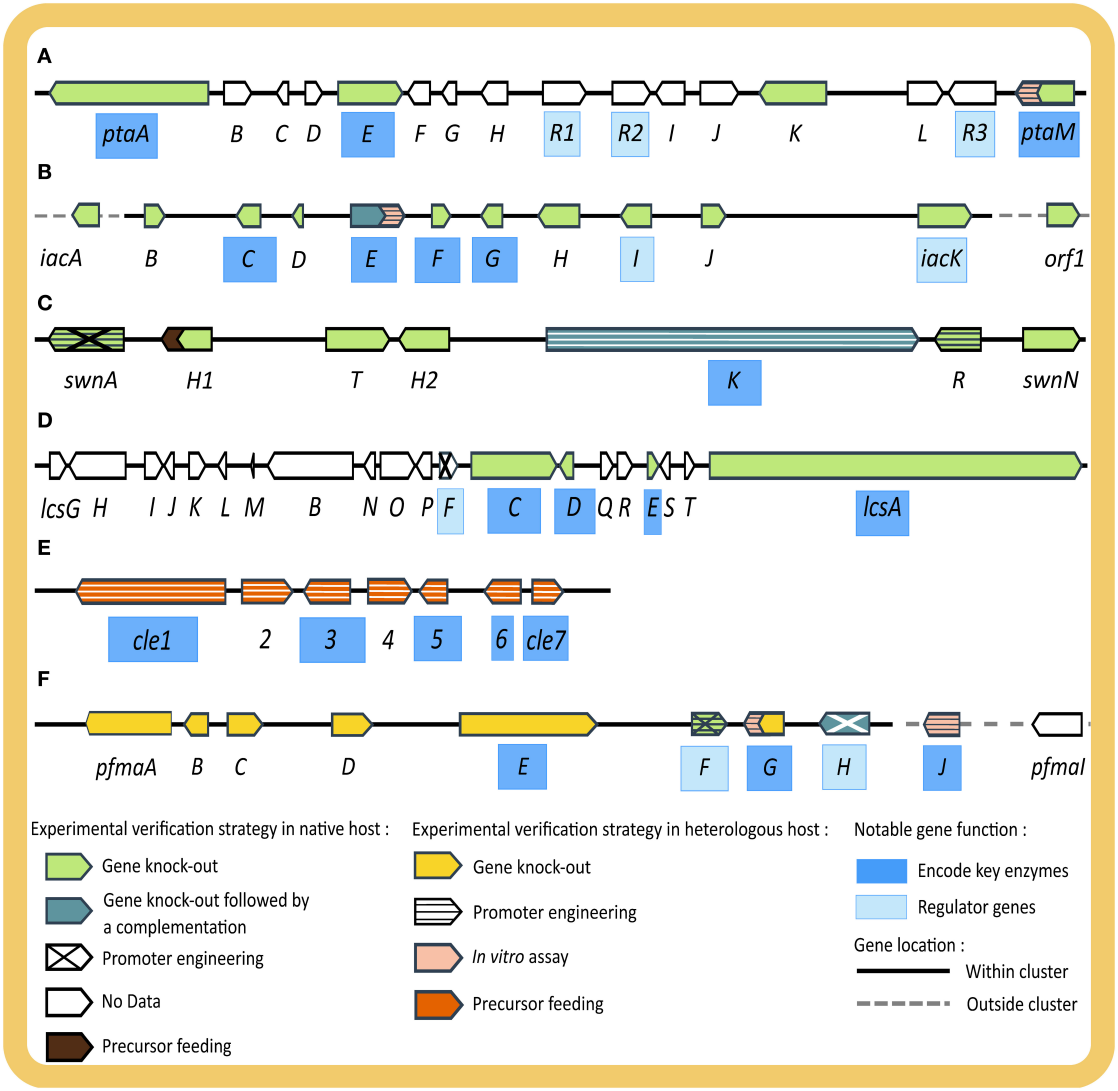
Illustration of four BGCs discovered in studies starting from phenotypic observation **(A–D)** and two BGCs from genotypic observation **(E,F)** including the experimental strategies used to verify and characterize their functions. **(A)** *pta* giving rise to pestheic acid (**20**), **(B)** *iac* encoding enzymes for isoA82775C (**21**), **(C)** *SWN* shown to be essential for swainsonine (**2**) production, **(D)** *lcs* giving rise to leucinostatin (**13**), **(E)** *cle* involved in the biosynthesis of chevalone E (**18a**) and its derivatives (**18b, 18c**), and **(F)** *pfma* essential for the production of 1,8 DHN (**25**) melanin. Genes are indicated as arrowheads, with names of genes encoding key enzymes highlighted in dark blue, (putative) regulatory genes in light blue. Fill and pattern of arrowheads depict experimental evidence (native host: light green—gene knock-out, dark green—gene knock-out followed by rescue via gene complementation, diagonal cross—promoter engineering, no pattern with white fill—no experimental data, brown—precursor feeding experiment; heterologous host: yellow—gene knock-out, horizontal stripe—promoter engineering, pink—*in vitro* assay, orange—precursor feeding experiment). Genes located within BGC are connected with a solid black line while genes outside the BGC with dotted gray line.

The first example starting from phenotypic observation is the elucidation of the biosynthetic pathway of chloropupukeananes in *P. fici* (Figures 3A,B). It started from the isolation of a novel secondary metabolite class with significant antitumor and anti-HIV activity, later named chloropupukeananins (**19**), by a bioassay-guided fractionation of culture extracts of *P. fici*, an endophytic fungus of tea plant (Liu et al., 2008). Based on retro-biosynthesis, two main precursors, pestheic acid (**20**) and isoA82775C (**21**), were proposed (Liu et al., 2008, 2009) and confirmed with a series of extensive experiments (Xu X. et al., 2014; Pan et al., 2018). In the 2014 study, Xu et al. hypothesized that a non-reducing PKS would be the key enzyme of the BGC of **20**, based on its structural similarity with other fungal diphenyl ethers. Homology-based genome mining was employed to find the gene encoding this enzyme from the genome of *P. fici*. The thus identified *pta* cluster was examined for the presence of genes encoding for putative tailoring enzymes in the pathway proposed by retro-biosynthesis. Transcriptional analysis by RT-qPCR demonstrated a correlation between increased transcript levels from the *pta* cluster (*ptaA, ptaM, ptaE, ptaH*) and an increase of **20** production, verifying the involvement of this BGC in the biosynthesis of **20**. Experimental verification was conducted by disrupting several genes including *ptaA, ptaE*, and *ptaM* in the native host, which resulted in abolishment of **20** and **19** production, whereas no function could be assigned to *ptaK* by gene disruption. PtaE was found to be the key phenolic coupling enzyme in the pathway. Heterologous expression of the key chlorinating enzyme PtaM in *E. coli* was performed to elucidate its biochemical mechanism and identify important intermediates (Xu X. et al., 2014). Since the production of the other precursor, **21**, was not affected by the disruption the mentioned key enzymes in the *pta* BGC, it was confirmed that a separate BGC was responsible for **21** production. In 2018, Pan et al. identified the *iac* BGC in the genome of *P. fici* based on gene homology and connected it to the biosynthesis of **21** by disrupting eight genes in the cluster, resulting in the loss of **21** and **19** production. This phenotype was successfully rescued with complementation of the key gene *iacE*, thus providing strong evidence for the involvement of the *iac* cluster in biosynthesis of **21**. Moreover, heterologous expression of the prenyl-transferase enzyme IacE in *E. coli* was also performed for enzymatic and kinetic study. Unexpectedly, gene deletion of *iacE* led the authors to the discovery of four new chloropestolides from *P. fici* (Pan et al., 2018). Genome sequencing, comparative genome analysis, and transcriptional analysis of BGCs from this organism were further reported by Wang et al. (2015), revealing the potential of *P. fici* as a prolific secondary metabolite producer.

The second example is the elucidation of the swainsonine BGC (*SWN*) (Figure 3C) across several studies in multiple endophytic fungi (Lu et al., 2016; Cook et al., 2017; Noor et al., 2020). Swainsonine (**2**) is an indolizidine alkaloid, originally observed in plants due to its toxicity to livestock, with potential applications in cancer therapy (Santos et al., 2011). It was originally isolated from several plant species but later found to be a fungal rather than a plant secondary metabolite (Cook et al., 2013). The first step toward pathway elucidation of swainsonine was taken in 2016, when Lu et al. sequenced the whole genome of *Alternaria* sect. *Undifilum* and proposed genes involved in the biosynthesis of **2** based on retro-biosynthesis and gene homology. In 2017, Cook et al. performed comparative genomics on the known producers of **2**, *Metarhizium robertsii, Slafractonia leguminicola* and *Alternaria* sect. *Undifilum*, and uncovered the *SWN* BGC (Cook et al., 2017). The authors searched for orthologous BGCs with a PKS gene that also carries an adenylation domain to load the pipecolic acid starter unit. The thus identified PKS gene was named *swnK* and its function was verified by gene disruption in *M. robertsii*, which resulted in abolishment of **2** production. In addition, the authors demonstrated a rescue of **2** production by homologous recombination using a complementary *swnK* gene. The amino acid sequence of the ketoacyl synthase domain of SwnK was later found to be essentially identical among all **2** producing *Alternaria* species (Noor et al., 2020). The genes adjacent to *swnK* in the genome were also analyzed by functional *in silico* evaluation to identify putative genes involved in **2** biosynthesis based on the retro-biosynthetic strategy (Cook et al., 2017). With this approach the authors predicted the full *SWN* cluster, including *swnN* and *swnR* (an Nmr-A like, and a nicotinamide dinucleotide-binding Rossman-fold reductase gene), *swnH1* (2-oxoglutarate-dependent oxidase gene), *swnH2* [Fe (II)-dependent dioxidase gene], *swnA* (aminotransferase gene), and *swnT* (transmembrane choline transporter gene). Extended comparative genomics were performed on published fungal genomes and identified the *SWN* in five different orders of filamentous Ascomycota from different ecological niches. Cultivation and secondary metabolite analysis of representatives of these orders, showed the presence of **2**, which indicates that swainsonine production is not a specialized trait of plant-associated fungi (Cook et al., 2017). In a recent study, the essential functions of *swnH1* and *swnH2* were confirmed by gene deletion in *M. robertsii*, whereas the *swnN, swnR* and *swnT* knock-out mutants still produced **2** (Luo F. et al., 2020). The role of SwnA in producing the pipecolic acid precursor for SwnK was confirmed by gene deletion and overexpression, however, it appears that pipecolic acid can also be produced by other (unconfirmed) pathways in *M. robertsii*, involving genes outside SWN BGC. Even after meticulous experiments, the exact role of SwnN, SwnR, and SwnT remain elusive. However, important pathway intermediates were identified, which allowed the conclusion that SwnH1 catalyzes the final step in **2** biosynthesis.

A third example is the pathway investigation and enhancement of the biosynthesis of perylenequinones, including Hypocrellin A (**16**), in the endophytic fungus *S*. sp. Slf14 from *Huperzia serrata*. These studies were started from the phenotypic observation of **16** with high pharmaceutical importance from an endophytic fungus *S. bambusicola* (Yang et al., 2009). Using *de novo* transcriptome assembly and retro-biosynthesis based on structural similarity of **16** with cercosporin, perylenequinones biosynthesis was suggested to involve the a type I PKS, an O-methyltransferase/FAD-dependent monooxygenase, an hydroxylase and another methyltransferase in *S. bambusicola* (Zhao et al., 2016). In order to further study the regulatory mechanisms of the PKS, the methyltransferase and hydroxylase genes, Liu et al. performed RT-qPCR of *S*. sp. Slf14 cultured under different Ca^2+^ concentrations, and in the presence of Ca^2+^ signaling antagonists (Liu et al., 2018). Under high Ca^2+^ conditions, perylenequinones production was enhanced, and the transcription dynamics of the putative pathway genes were similar to those of the known Ca^2+^ sensors *cam, cna*, and *crz1*. Later on, the same pathway genes were pinpointed by transcript analysis in *S. bambusicola* S4201, and genes encoding a putative O-methyltransferase/FAD-dependent monooxygenase, an Flavin-dependent oxidoreductase, a multicopper oxidase and a Zink-finger transcription factor (Li et al., 2019). Recently, comparative transcriptomics were used to study the transcription of the genes in the proposed BGC in *S*. sp. Slf14 grown with different carbon sources (Liu et al., 2020). The highest perylenequinones production was observed with fructose and the transcription of the putative perylenequinones biosynthetic genes including the Zink-finger transcription factor, and important enzymes in precursor supply were upregulated, whereas competing pathways, such as fatty acid synthesis were downregulated. This could be attributed to the activity of global regulators, e.g., Cre1, PaC (upregulated in presence of fructose) and LaeA (downregulated) (Liu et al., 2020). To summarize, even though the whole genome sequence of *S*. sp. Slf14 was obtained (Yang H. et al., 2014), no successful gene deletion of the putative BGC has been reported to date that would unambiguously confirm the importance of the BGC for **16** production. Nevertheless, many years of extensive work have provided convincing evidence for the function of this BGC and remarkable insight into the regulation of perylenequinones biosynthesis.

The fourth example is reported by Wang et al. (2008) in the re-isolation of radicicol (**3a**) from the endophytic fungus *C. chiversii* and the elucidation of its biosynthetic pathway. **3a** is a known fungal polyketide with prospective anticancer activity, and was originally isolated by a bioassay-guided fractionation based on its inhibitory activity against Hsp90 (Turbyville et al., 2006). Based on the similarity of its scaffold with fungal Resorcylic Acid Lactones (RALs), retro-biosynthesis led to the hypothesis that a highly reducing PKS (hrPKS) and a non-reducing PKS (nrPKS) would form the core structure. A fosmid library of the genome was constructed and screened for the putative biosynthetic genes using degenerate PCR primers designed with the sequence of a close homolog. In the sequence of the fosmid hits, a putative BGC encoding the expected PKS enzymes and a number of tailoring enzymes, were identified. Next, targeted disruptions of the core biosynthetic genes, namely the *ccRads1* (hrPKS), *ccRads2* (nrPKS), and radR (a gene encoding a putative positive transcriptional regulator) in the native host led to a loss of **3a** production. The disruption of one of the putative tailoring enzymes, RadP, led to the accumulation of Pochonin D (**4**), now shown to be a pathway intermediate in **3a** and **3b** biosynthesis (Wang et al., 2008).

In the fifth example, *Purpureocillium lilacinum*, an endophyte with a well-known biocontrol use against various plant pathogens in agriculture, was shown to be a prolific producer of leucinostatins (**12**) (Wang et al., 2016), which are peptaibiotics with a wide range of activities including, antibiotic (Fukushima et al., 1983) and antitumor effects (Kawada et al., 2010). Based on BGC homology and a retro-biosynthesis approach, Wang et al. hypothesized that the *lcs* gene cluster involving 20 genes would be responsible for biosynthesis of **12**. From there, gene knock-outs on *lcsC, lcsD, lcsE*, and *lcsA* were performed by homologous recombination in the native host to verify their function in the pathway (Figure 3D). In addition, the OSMAC strategy coupled with RT-qPCR and RNAseq analysis was employed to determine the boundaries of the BGC. Lastly, the overexpression of a cluster specific regulator, LcsF, rounded up the study by confirming the co-regulation of the genes in the newly identified BGC.

In the sixth example, BGC homology and retro-biosynthesis were applied to propose a biosynthetic pathway for nodulisporic acids, bioactive indole diterpenes produced in the endophytic fungus *Hypoxylon pulicicidum* strain MF5954, from *Bontia daphnoides* (Nicholson et al., 2018). To confirm the involvement of the genes in the proposed gene cluster (NOD), Van De Bittner et al. (2018) used a multigene assembly strategy to reconstitute parts of the biosynthetic pathway in the heterologous host *Penicillium paxillin*. Thereby, they characterized the function of four genes involved in the biosynthesis of the noludisporic acid core compound, noludisporic acid F (**23**).

Although the above-mentioned elucidation efforts of several genes in a BGC offer comprehensive insight into the biosynthesis of secondary metabolites in endophytic fungi, studying a single yet specific biosynthetic enzyme can be promising for bioprospecting as well. In this regard, a success story was reported for *Hypoxylon* sp., which was discovered to produce a series of volatile organic compounds, including 1,8-cineole (**24**), a monoterpene with high commercial and pharmaceutical value (Tomsheck et al., 2010). Since up to that point, no fungal monoterpene synthases were known, *Shaw* et al. selected eight putative terpene synthase genes from the whole genome sequence of the fungus based on sequence homology to plant terpene synthase genes. They expressed these genes heterologously in *E. coli* and analyzed the product range (Shaw et al., 2015). In this way, they identified the first fungal monoterpene synthase Hyp3 and established efficient production of **24**. Kinetic studies, homology modeling and site directed mutagenesis identified the crucial active site residues for catalysis and substrate selectivity of Hyp3. Taken together, this extensive study was an important step in the bioprospecting of an endophytic fungal enzyme with potential for industrial application.

### Studies Starting From Genotypic Observation

In the first example starting from genotypic observation, Wang et al. (2019) successfully identified the plausible biosynthetic pathways of Chevalone E (**18a**) and its novel analogs by studying the genome of the endophytic fungus *A. versicolor* 0312 (Figure 3E). First, WGS, assembly, and annotation were conducted. Since hybrid polyketide-diterpenoid (PK-DT) scaffolds are often part of molecules with a wide range of bioactivities and only few BGCs giving rise to this scaffold were known at the time, this study was aimed to accelerate the discovery of interesting novel molecules from this scaffold. The authors performed rule-based genome mining based on BGC homology. Initially, a BLAST search was performed to find a genomic region that encodes both a PKS and a geranylgeranyl pyrophosphate synthase, and the genome neighborhood was analyzed for putative tailoring enzymes, resulting to the discovery of a proposed *cle* BGC. Given that the *cle* cluster was significantly different from known PK-DT hybrid clusters, it was deemed an interesting target. PCR amplification was used to clone the target genes for expression in a heterologous host, *A. oryzae*, for experimental verification. Several gene combinations were tested to study the function and order of all enzymes in the pathway, as well as the intermediates produced. Lastly, the authors designed a combinatorial pathway by co-expressing parts of the *cle* cluster with the terpene synthase of the homologous *sre* cluster from *A. felis* 0260 to obtain novel molecules (**18b** and **18c**). These were seen to enhance the efficacy of doxorubicin against breast cancer cells. More importantly, these molecules possess a characteristic five-membered lactone ring, which is very rare in meroterpenoids and has never been seen in fungal meroterpenoids (Wang et al., 2019).

The second example started from the discovery of a cryptic BGC, encoding for enzymes later found to produce 1,8-dihydroxynaphthalene (DHN) (**25**) melanin in *P. fici* (Figure 3F) (Zhang et al., 2017, 2019). DHN melanin plays significant roles in secondary metabolite production, UV protection, oxidative stress and pathogenesis of fungi (Eisenman et al., 2020). The study aimed at identifying fungal pigments and their physiological roles and the authors searched for PKS genes, typically involved in melanin biosynthesis (Zhang et al., 2017). Based on sequence homology the authors identified the putative PKS gene *pfmaE* in the genome of *P. fici*. Deletion of this gene showed a complete abolishment of melanin biosynthesis, as seen by decreased pigmentation and incomplete spore development, which were later rescued by feeding of pathway intermediates. However, since phenotyping efforts with different extraction methods and analyses by HPLC and LC-MS analysis could not distinguish differences in the chemical profile of wild type and *pfmaE* mutant due to the low concentration or cryptic nature of melanin biosynthesis, the authors performed further experimental verification by heterologous expression of the predicted *pfma* cluster (*pfmaA-D, G, H*+*E*) in *A. nidulans*. Metabolite profiles of several recombinant strains were compared to assign enzyme functions and identify intermediates. Prominent intermediates of the melanin pathway, T4HN (**26**), which is unstable and easily oxidized to form flaviolin (**27**), and scytalone (**28**) were observed in the strains expressing p*fmaE*, while they were absent in a strain only expressing the other p*fma* genes, thus suggesting the contribution of PfmaE in the pathway. To further study the function of each gene in the cluster, gene deletions in the p*fma* expressing heterologous host were performed and only PfmaE and G were found to be essential for the formation of the pathway intermediates. In conclusion, the authors suggested that PfmaE produces **26** which is reduced by PfmaG to form **28**. Based on homology search, the authors suggested PFICI_02498 as syctalone dehydratase or PfmaD as vermelone dehydratase to be responsible in the final steps of melanin biosynthesis. However, experimental verification was not done on those genes. Co-expression of a putative regulatory gene, *pfmaF*, was performed which resulted in a significant increase of **26–28** in the heterologous host, indicating an involvement in the melanin biosynthetic pathway. Since the function of two genes encoding transcription factors in the *pfma* cluster, *pfmaF* and *pfmaH* were not clearly characterized in the first study, a follow up experimental verification using gene deletion in the native host was conducted (Zhang et al., 2019). With transcriptomic analysis and chemical studies, the authors showed the cluster-specific role of PfmaH in the melanin biosynthetic pathway. Another transcription factor, PfmaF, was verified to be a global regulator of secondary metabolite biosynthesis in *P. fici*, including its role in assisting PfmaH in promoting melanin synthesis. The function of other genes was also verified in this study by *in vitro* characterization, namely PfmaJ previously named PFICI_02498 as scytalone dehydratase and PfmaG or PFICI_05460 as T4HN reductase. In another study, a second global regulator, RsdA, was also reported to be involved in melanin biosynthesis of *P. fici*. Experimental verification by gene deletion showed a remarkable increase in melanin production, which was suggested to be achieved by its modulation of PfmaE PKS expression (Zhou S. et al., 2019).

As studying the entire genes in a pathway might be difficult considering their complexity and the big size of BGCs, some studies focus on ascertaining the presence of key genes encoding the enzymes which could serve as markers in the pathway. As an example, a the *ssars* gene encoding 5-alk(en)ylresorcinol synthase was identified in the genome of *S*. sp. Slf14, an endophytic fungus from *Huperzia serrata* based on sequence homology to known Type 3 PKSs (Yan et al., 2018). Yan et al. studied the gene in two heterologous host, *E. coli* and *S. cerevisiae* by cloning the intron free DNA of *ssars* through reverse transcriptase PCR. Substrate feeding with fatty acids showed the potential of the enzyme to generate a wide range of fatty acid primed resorcinol derivatives, including **14**, using the exogenously supplied precursors in the heterologous hosts (Yan et al., 2018). With this strategy, bioprospecting efforts can be efficiently directed to the use of specific enzymes in the production of secondary metabolites with biopharmaceutical importance.

Despite the great potential of starting a study from genotypic observation, successful stories from this approach are much less prevalent than when starting from phenotypic observation. In the next section of this review, we will provide some insight into the major hurdles in the field.

## Challenges in Genotyping of Endophytic Fungi

Compared to other microorganisms, such as bacteria, the genotyping effort of endophytic fungi appears to be lagging behind. This may be attributed to the fact that genomics of fungi in general is still in its infancy and that the currently available BGCs in the databases might be of low predictive value for fungal BGCs (van der Lee and Medema, 2016).

Even though technologies of next generation sequencing (NGS) have changed dataflow dramatically as the amount of sequencing data is growing at a significant pace (Goodwin et al., 2016), only a miniscule fraction of endophytic fungi have their whole genomes sequenced and published. From a thorough data base search (NCBI whole genome sequences and pubmed), we have compiled a list of publicly accessible WGS projects of proven endophytic fungi (Table 3). Based on a search of the JGI project database and the Mycocosm (JGI, 2019), we believe that many more genomes will be published in the near future, but certainly not as many as one would hope for. This is most likely due to the uncultivability of many endophytic fungi outside their habitat, which impedes their isolation process (Rashmi et al., 2019). Thus, in the future directions section we will discuss several advances in environmental genomics that we think will have a positive impact on the field of endophyte biology in general, and in particular fungal genomics.

**Table 3:** List of endophytic fungi subject to whole genome sequencing projects referenced in the literature.

**Endophytic fungus**	**Host plants**	**Accession number**	**Observed bioactive secondary metabolite**	**Activity**	**References**
*Alternaria* sp. MG1	*Vitis vinifera*	QPFE00000000^a^	Resveratrol	Antitumor, cardioprotective, antioxidant, anti-inflammatory	Lu et al., 2019
	*Vitis quinquangularis*	NA^b^	(±)-Alternamgin	Antiproliferative	Wu J. C. et al., 2019
*Alternaria* sect *Undifilum*	*Oxytropis kansuensis*	NA^b^	Swainsonine	Anticancer	Lu et al., 2016
*Amphirosellinia nigrospora* JS-1675	*Pteris cretica*	SCHM00000000^a^	Oxygenated cyclohexanone	Antimicrobial	Jeon et al., 2019; Nguyen et al., 2019
*Aspergillus versicolor* 0312	*Paris polyphylla* var. *yunnanensis*	NA^b^	Chevalone E	Anticancer	Wang et al., 2019
*Ascocoryne sarcoides*	*Eucryphia cordifolia*	AIAA00000000^a^	ND	ND	Gianoulis et al., 2012
*Ascomycete* sp F53	*Taxus yunnanensis*	KT874412^a^	Lijiquinone 1	Anticancer and Antifungal	Cain et al., 2020
*Cadophora* sp. DSE1049	*Salix rosmarinifolia*	PCYN00000000^a^	ND	ND	Knapp et al., 2018
*Colletotrichum gloeosporioides* Cg01	*Huperzia serrata*	QRFY00000000^a^	Huperzine A	Memory enhancer	Kang et al., 2019
*Colletotrichum truncatum*	*Glycine max*	VUJX00000000^a^	ND	ND	Rogério et al., 2020
*Dactylonectria torresensis* BV-349	*Solanum nigrum*	VYKH00000000^a^	ND	ND	Gramaje et al., 2020
*Dactylonectria torresensis* BV-666	grapevine rootstock 110 Richter	VYKG00000000^a^	ND	ND	Gramaje et al., 2020
*Daldinia childiae* JS-1345	*Abies koreana*	VYXO00000000^a^	ND	ND	Kim et al., 2020
*Diaporthe ampelina*	*Commiphora wightii*	LWAD00000000^a^	ND	ND	Bhargavi et al., 2018
*Diaporthe* sp. *HANT25*	*Hydnocarpus anthelminthicus* Pierre ex Laness	JACBFG000000000^a^	Mycoepoxydiene	Anticancer	Tulsook et al., 2020
*Falciphora oryzae*	*Oryza granulata, O. sativa*	JNVV00000000^b^	Indole derivatives	Plant growth factor	Sun et al., 2020
*Fusarium solani* JS-169	*Morus alba*	NGZQ00000000^a^	Red to purple pigments	Antimicrobial	Kim et al., 2017b
*Fusarium tricinctum* T6	*Taxus baccata wallichiana*	PTXX00000000^a^	Lateropyrone	Antibacterial	Akone et al., 2016; Meena et al., 2018
*Gaeumannomyces* sp. JS-464	*Phragmites communis*	NGZR00000000^a^	C-glycosylated dialkylresorcinol derivatives and glycosylated anthraquinone	Anti-inflammatory	Kim et al., 2017a
*Hirsutella minnesotensis* 3608	*Hirsutella glycines* Ichinohe	JPUM00000000^a^	ND	ND	Lai et al., 2014
*Hypoxylon* sp. E7406B	*Clarisia racemosa*	JYCQ00000000^a^	1,8-cineole	Essential oil	Shaw et al., 2015
*Hypoxylon* sp. CI-4A	Barley seeds	MDGY00000000^a^	ND	ND	Wu et al., 2017
*Hypoxylon* sp. EC38	Barley seeds	MDCK00000000^a^	ND	ND	Wu et al., 2017
*Hypoxylon* sp. CO27	Barley seeds	MDCL00000000^a^	ND	ND	Wu et al., 2017
*Hypoxylon pulicicidum* MF5954	*Bontia daphnoides*	PDUJ00000000^a^	Nodulisporic acids	Insecticide	Nicholson et al., 2018
*Metarhizium robertsii* ARSEF 23	ND	ADNJ00000000^a^	Swainsonine	Anticancer	Gao et al., 2011; Cook et al., 2017
*Metarhizium robertsii* ARSEF 2575	ND	0414787^d^	Swainsonine	Anticancer	Cook et al., 2017
*Mucor endophyticus* CBS 385–95	*Triticum aestivum*	PRJEB30975^b^	ND	ND	Lebreton et al., 2020
*Paraphaeosphaeria sporulosa*	*Fragaria × ananassa*	LXPO00000000^a^	Diketopiperazine	Antibiotic	Carrieri et al., 2020
*Penicillium aurantiogriseum* NRRL 62431	*Corylus avellana*	ALJY00000000^a^	Paclitaxel	Anticancer	Yang et al., 2014
*Penicillium chrysogenum*	*Fagonia cretica*	MWKT00000000^a^	Hypocrellins	Antifungal	Meng et al., 2011; Ding et al., 2020
*Penicillium brasilianum* LaBioMMi 136	*Melia azedarach*	LJBN01000000^b^	Meroterpenoids	Anti-inflammatory and Antibacterial	Fill et al., 2018
*Penicillium citrinum JCM 22607*	*Citrus reticulata*	BCKA00000000^a^	Polyphenol and flavonoid	Antioxidants	Mefteh et al., 2018
*Penicillium citrinum* DSM 1997	*Citrus reticulata*	LKUP00000000^a^	Citrinin	Antibiotic	Schmidt-Heydt et al., 2019
*Penicillium dangeardii*	*Lysidice rhodostegia*	NA^b^	Azaphilones	Antiproliferative, Anti-inflammatory, and Antioxidant	Wei et al., 2020
*Penicillium polonicum* hy4	*Huperzia serrata*	QPIC00000000^a^	Huperzine A	Alzheimer treatment	Kang et al., 2019
*Periconia macrospinosa* DSE2036	*Festuca vaginata*	PCYO00000000^a^	ND	ND	Knapp et al., 2018
*Pestalotiopsis fici* CGMCC3.15140	*Camilia sinensis*	ARNU00000000^a^	Chloropupukeananins	Antimicrobial, Antitumor, and Anti-HIV activities	Wang et al., 2015
			Ficiolide K	ND	Wu et al., 2016
			Pestaloficins	ND	Zheng et al., 2017
*Phialocephala scopiformis* DAOMC 229536	*Picea glauca*	LKNI00000000^a^	Rugulosin	Anti-insect	Walker et al., 2016
*Phyllosticta capitalensis* Gm33	*Citrus sinensis (L.) Osbeck*	QXPW00000000^a^	ND	ND	Rodrigues et al., 2019
*Phyllosticta capitalensis* LGMF01	*Citrus latifolia*	LOEO00000000^a^	ND	ND	Rodrigues et al., 2019
*Phyllosticta capitalensis* CBS 128856 v1.0	*Citrus* spp.	1109085^c^	ND	ND	Guarnaccia et al., 2019
*Phyllostricta citribraziliensis* CBS100098	*Citrus* spp.	1109089^c^	ND	ND	Guarnaccia et al., 2019
*Pochonia chlamydosporia*	*Musa acuminata*	AOSW00000000^a^	ND	ND	Larriba et al., 2014
*Pseudofusicoccum stromaticum*	*Myracrodruon urundeuva*	NA^b^	Cyclopeptide and Rotenoids	Anticancer	Sobreira et al., 2018
*Purpureocillium lilacinum*	ND	LSBH00000000^a^	Leucinostatins	Antibiotic and Antitumor	Wang et al., 2016
*Rhodotorula graminis* WP1	*Populus trichocarpa*	JTAO00000000^a^	Phytohormones	Promoting plant growth	Firrincieli et al., 2015
*Sarocladium brachiariae* HND5	*Brachiaria brizantha*	RQPE00000000^a^	ND	ND	Yang et al., 2019
*Serendipita indica* DSM 11827	*Arabidopsis thaliana*	CAFZ00000000^a^	ND	ND	Zuccaro et al., 2011
*Shiraia* sp. Slf14	*Huperzia serrata*	AXZN00000000^a^	alk(en)yl-resorcinol polyketides	Antibacteria	Yang H. et al., 2014; Yan et al., 2018
			Perylenequinones	Anticancer, antibacterial, antiviral, and memory enhancer	Liu et al., 2018, 2020
*Taxomyces andreanae* CBS 279.92	*Taxus brevifolia*	ALYI00000000^a^	ND	ND	Heinig et al., 2013
*Xylona heveae* TC161	*Hevea brasiliensis*	JXCS00000000^a^	ND	ND	Gazis et al., 2016

*The columns contain the following information: fungal strain, host plant, accession number with the respective source database indicated by footnote, observed bioactive secondary metabolites and their bioactivity, and primary literature reference. All entries were assembled at contig or scaffold level*.

*ND, no data*.

a *NCBI*.

b *Not published, only mentioned in literature*.

c *JGI*.

d *REEIS*.

In terms of genome mining and BGC discovery, several hurdles remain to be overcome when studying endophytic fungi. The first problem is the low predictive value of known BGCs for the annotation of fungal BGCs in bioinformatic tools with a conventional rule-based approach. A global analysis of BGCs in the genomes of 24 *Penicillium* species found that only 16% of the PKS- and NRPS-derived BGCs can be connected to a pathway and the respective secondary metabolites, while the rest have no predictive value for secondary metabolite (Nielsen et al., 2017). The prediction of fungal BGCs is problematic since most of the bioinformatic tools were developed for bacterial genomes, which have a different architecture: fungal BGCs are more often split over multiple loci and/or have a bidirectional orientation in which the cis-regulatory region is shared between a set of genes (van der Lee and Medema, 2016). Beyond that, fungi possess a large number of BGCs lacking even the homologs of scaffolding enzymes, so-called exotic BGCs, which are even more difficult to annotate (van der Lee and Medema, 2016). In order to solve such annotation problems, transcriptomic studies are often employed in other organisms, e.g., bacteria and plants, however, since many of these genes are also transcriptionally silent under experimental conditions this technique is often not applicable, which makes them frequently misannotated (van der Hooft et al., 2020). Similar to bacterial BGCs, plant biosynthetic genes are often also unsuitable for comparative analysis to predict endophytic fungal BGCs. Many studies propose that the biosynthetic pathways in endophytic fungi are most likely different from those in their host plants (Kusari et al., 2009b; Yang et al., 2014; Kang et al., 2019; Qiao et al., 2020) even though lateral gene transfer from plant to endophytic fungi was originally hypothesized. Two prominent examples for molecules present in fungi and plants yet formed by different pathways are gibberellins and indole acetic acid (Salazar-Cerezo et al., 2018). Taken together, the prediction of fungal BGCs is at high risk of being inaccurate with the conventional rule-based approach.

Even in case of successful functional annotation of BGC boundaries and all the contained genes, it remains challenging to predict the structure of the secondary metabolites produced. Unlike studies starting from phenotypic observations with the elucidated chemical structure in hand, judging the biological or biotechnological relevance of a predicted secondary metabolite purely based on the genes encoded in a BGC is nearly impossible to date (Weber and Kim, 2016; van der Hooft et al., 2020). Platforms like SANDPUMA (Chevrette et al., 2017), or a combination of individual module-level prediction tools like antiSMASH (Medema et al., 2011) and PRISM (Skinnider et al., 2017) offer various algorithms to predict full chemical structures, yet often lead to a massive amount of combinatorial possibilities with error-prone results, especially for unusual and rare modifications from less studied organisms, like endophytic fungi. In addition, an effective method to sort the prediction results based on their match rate with the actual structures has not been reported (van der Hooft et al., 2020).

Despite the mentioned challenges, genome mining allows to explore cryptic and novel BGCs. Current drawbacks are mainly due to the early stages of genetics and genomics compared to the phenotyping methods. As reviewed in the next section, we expect that some of these problems will be overcome with the availability of more functional genomics studies in fungi and especially endophytic fungi.

## Future Directions for Successful Secondary Metabolite Bioprospecting From Endophytic Fungi

Despite the current shortcomings and limitations in the field, we observe some emerging technologies and tools that might advance the bioprospecting effort of endophytic fungi in the future. In this last section we will review these developments within three major categories: (1) the cultivation and isolation process, (2) culture-independent techniques, and (3) genome editing techniques.

### Advanced Cultivation and Isolation of Environmental Isolates

Studying endophytes in axenic experimental conditions is highly challenging considering the absence of metabolites and stimuli provided by the host plant and other members of the microbial community. It appears that the lack of essential, life-supporting factors leads to the majority of endophytic fungi being uncultivable under experimental conditions and hampering their isolation process. Currently we have no reliable information on the ratio of cultivable to uncultivable fungal endophytes, but environmental DNA surveys suggest that the diversity of species is far greater than the diversity of cultivated isolates (Wu B. et al., 2019). A study on the endophytic fungal community of *Dysphania ambrosioides*, found that almost 10 times more species can be identified by amplicon sequencing than can be isolated by cultivation (Parmar et al., 2018). In order to increase the number of cultivable species and study their biosynthetic potential, it is of utmost importance to improve cultivation and isolation procedures. Next to the technological advances discussed in the following sections, closer collaboration of bioprospectors with and training from experienced mycologists would also help solve the cultivability problem.

#### FIND Technology

Recently, Libor et al. (2019) developed a Fungal one-step IsolatioN Device (FIND) which allows the isolation of rare fungi from terrestrial and marine samples. This technology is an adaptation of the Isolation chip (iChip), originally developed to grow bacteria from environmental samples using the naturally occurring nutrients and growth factors from their own habitat (Berdy et al., 2017). Both technologies allow for high throughput isolation of microbes by utilizing several hundred miniature diffusion chambers in a growth device, each inoculated with a single cell (environmental isolates or engineered variants of a single strain). This growth device is brought back into its natural environment (e.g., submerged in sea water or buried in damp soil) to allow for diffusion of nutrients and signaling molecules from the environment and between the microbes in the individual chambers. This setup facilitates the cultivation of novel species without the need to optimize artificial growth conditions and allows for capturing broader biological diversity. The use of this technology has not been reported for plant-associated fungi, but a so-called “rhizochip” was developed to study bacteria in the rhizosphere of the canola plant (Gurusinghe et al., 2019). For the study of epiphytes, living on the surface of a plant, we could imagine that techniques to attach a FIND to a plant could be developed. However, developing a similar system for endophytes, where an incision on a leaf, the stem or bark would be required, appears challenging. An easier strategy could be to use the FIND submerged in a plant liquid culture or in media spiked with plant exudates. Clearly, there is still a lot of room for innovation in this field of research, but we think that facilitating the isolation and cultivation process of endophytic fungi would really accelerate the possibilities to mine for secondary metabolites and biosynthetic genes.

#### Microfluidic Devices

Microfluidic platforms have been used as a powerful approach to evaluate the responses of plant cells to external perturbations, such as nutrients, temperature, light and other abiotic or even biotic factors like a pathogen (Meier et al., 2010; Busch et al., 2012; Jiang et al., 2014). It has a higher throughput compared to conventional cultivation techniques and a more precisely controlled environment. However, to date only a few studies use this technology to study biotic interactions of plants with their symbionts. A recent breakthrough using this technology was reported by researchers at the U.S. Department of Energy's Argonne National Laboratory. They successfully developed a Root Microbiome Interaction (RMI) chip to study the interaction between *Populus tremuloides* (aspen tree) and its bacterial root microbes (Noirot-Gros et al., 2020). The microfluidic setup in the RMI-chip is coupled with advanced live imaging microscopy to allow for direct visualization of the dynamic interactions at cellular resolution at real-time. However, it only enables the observation of the root, which is relatively easy since it can be immersed and continuously perfused to monitor the biochemical interactions. Studying other plant parts is not as easy since the system needs to facilitate gas exchange and light exposure.

Since bacterial colonization is significantly different from fungal, optimization of these microfluidic devices is necessary to study fungal endophytes. Advances in microfluidic devices for fungi were recently reviewed (Zhou W. et al., 2019) but we'd like to highlight one chip design recently published by Millet et al. in 2019: a ready-to-use microfluidic device with chambers and boundary channels forming the letters “ORNL” was established to study binary interactions of various organisms, e.g., a plant-growth promoting bacterium and the fungus *Mortierella elongata* NVP64+, and *Nicotiana attenuata* seedlings and the fungus. In these binary interactions it was observed that the microbes did not influence each other strongly, although a small part of the bacterial population showed altered and previously unknown cell morphologies. With the plant-fungus co-culture, however, clear colonization of the plant roots by the endophyte was observed (Millet et al., 2019). The ease of use of these ready-to-use ORNL devices might make them widely applicable to study endophytic fungi and their BGCs in the presence of their host plants and allow for the tracking of their dynamics at high spatial and temporal resolution. Given that plant development from a single protoplast has been studied in microfluidic devices before (Sakai et al., 2019), we could even imagine using a similar setup to study the reciprocal influence of microbiota and plants with such devices. This would be particularly interesting for aerial parts of plants rather than roots and might allow for downstream metabolomics analysis and secondary metabolite discovery.

### Culture Independent Techniques to Study Microbial Communities

Bioprospecting of natural products is evolving from studying the biosynthetic potential of isolated organisms to a more comprehensive approach of studying microbial communities as a whole (meta-omics) or individual organism without prior isolation by cultivation (single-cell -omics). The meta-omics strategy has been reviewed in detail recently (van der Hooft et al., 2020) but we will highlight some developments specific to fungi. Single-cell analysis of fungi is still in its infancy and we will give a broader perspective on where we see the field going.

#### Meta-Omics of Fungal Communities

With decreasing cost of sequencing and advanced computational analysis pipelines, shotgun metagenomics has become an important technique to study microbial communities (Pérez-Cobas et al., 2020), particularly for uncultivable species. With sufficient sequencing depth, shotgun metagenomics has been shown to provide high quality assemblies of BGCs (Kim et al., 2012; Bengtsson-Palme et al., 2014; Donia et al., 2014; Sugimoto et al., 2019; Youngblut et al., 2020), entire chromosomes or even whole genomes from a microbial community (Chen et al., 2020). Unfortunately, to date many shotgun metagenomics projects are focused on bacteria and exclude fungal DNA in the sample preparation stage, thereby limiting the number of useful datasets for researchers interested in fungal BGCs. Donovan et al. have, however, developed a computational pipeline to identify fungal genes/contigs in metagenomic datasets with their FindFungi pipeline and identified several fungi in animal-associated microbiomes (Donovan et al., 2018). Several other tools that are not specific to fungi but can be applied to identify bacterial and eukaryotic contigs from metagenomic samples have been developed and recently compared in terms of performance (Ye et al., 2019). The most recent one is worth mentioning since it is an extension of the highly performant MMSeqs2 platform (Mirdita et al., 2020) that has gained significant traction in the environmental genomics field over the last few years (Steinegger and Söding, 2017; Santos et al., 2020; Segawa et al., 2020). As discussed by Ye et al. (2019), all of the tools heavily rely on reference genomes in public data bases and are therefore error-prone in undersampled branches of the tree of life. MMSeqs2 taxonomy appears to fare much better than its competitors in this regard, since it cross-validates its hits and thereby finds the last common ancestor of the query sequence (Mirdita et al., 2020). Another common problem in metagenomics analysis is the contamination of reference genomes with contigs that were incorrectly assigned to the respective species. Also here the developers of MMSeqs2 have recently developed a tool to “clean-up” these contaminations and therefore improve the quality and reliability of the database entries (Steinegger and Salzberg, 2020). With these improved reference databases and with the most recent tools, the analysis of existing and new metagenomic datasets will be dramatically improved and hopefully yield many interesting fungal contigs for exploration.

Another promising strategy is the use of long-read sequencing technologies, i.e., Pacific Bioscience or Oxford Nanopore sequencing on metagenomic libraries. Despite the relatively high error rates observed in these technologies, in particular Oxford Nanopore has been shown to provide excellent recovery of functional genomic units, entire chromosomes, and whole genomes of bacteria (Tyler et al., 2018; Arumugam et al., 2019; Moss et al., 2020) and fungi (Dutreux et al., 2018; Liu et al., 2019; McKenzie et al., 2020). Recent developments in real-time selective sequencing also allow for the depletion or enrichment of specific sequences during the sequencing run (Loose et al., 2016; Edwards et al., 2019; Masutani and Morishita, 2019; Kovaka et al., 2020; Payne et al., 2020). One could imagine that this strategy could be applied to suppress the sequencing of highly abundant species in a microbiome and thereby enhance the recovery of species with lower abundance.

In addition to these culture-independent genomics strategies, meta-transcriptomics and meta-metabolomics can be employed to give a more comprehensive view of a microbial community, since they allow for the analysis of gene expression levels and biochemical profiles thus revealing the details of the metabolic status of a community within a specific time frame. Please refer to van der Hooft et al. (2020) for a review on the emerging strategies and tools used to integrate metagenomics and metabolomic datasets. One of the biggest challenges of multi-omics strategies is the large quantity of data produced, which makes data integration a daunting task. Refinement and standardization of data structures might be a solution to allow the programmatic access by the different tools, but the application is yet to be seen in the field. Despite its great opportunities, there is to our knowledge no study applying meta-omics to study endophytic fungi. There is one very recent study integrating holobiont metabolomics with amplicon sequencing of the endophytic fungi of separated plant parts of *Ephedra sinica* to correlate the microbiome composition with the metabolite profiles (Zhang et al., 2020). Since culture-independent techniques are inherently high-throughput strategies and allow for the study of uncultivable organisms, we expect to see many more studies soon that lead to the discovery of novel BGCs and secondary metabolites.

#### Single-Cell -Omics of Microbial Communities

Single-cell -omics aims to gather information from a single cell of an organism, or an individual microbial cell from a community. In the latter case, this allows for the study of uncultivable organisms, and/or community members of low abundance. Herein, single-cell genomics is the most advanced of the -omics techniques and has even yielded near-complete genome assemblies of environmental microbes (Seeleuthner et al., 2018; Pachiadaki et al., 2019; Dam et al., 2020). In this technology, sorting of cells by flow cytometry is used for single cell isolation, then multiple displacement amplification is conducted followed by sequencing. Although this technology rarely yields full genome coverage, it offers a bright future to facilitate genomic exploration of unculturable organisms (Woyke et al., 2017). A recent study on bacterial environmental samples even suggests that the recovery of genomes with the single-cell approach is higher than that of a shotgun metagenomics approach (Dam et al., 2020). The major difficulty in using this technology on endophytic fungi is probably the preparation of single cells. The two studies applying single-cell genomics to fungi used spores that can easily be harvested from cultivable fungi (Ahrendt et al., 2018; Montoliu-Nerin et al., 2020). However, for uncultivable organisms this is not possible and other strategies need to be developed. Looking at the recent advances in plant single-cell genomics (Luo C. et al., 2020), the most promising strategies, will most likely be the preparation of protoplasts by cell wall digestion (host plant and fungal cells need to be digested), and/or the isolation of nuclei (Montoliu-Nerin et al., 2020). Once a suspension of cells or nuclei is prepared, they need to be separated by manual sorting or by Fluorescence-activated cell sorting (FACS). Depending on the presence of suitable markers for staining needed for FACS analysis and the overall numbers of harvested cells or nuclei, one strategy might be better than the other. It appears that in the plant community manual sorting is more common at this stage than FACS (Luo C. et al., 2020) and this might also turn out to be the better strategy for endophytic fungi. However, since flow cytometry of filamentous fungi has been demonstrated (Bleichrodt and Read, 2019), it is possible that such a high-throughput approach might become applicable for the sorting of fungal endophytes.

Recent advances in animal single-cell technologies have also allowed for multimodal single-cell -omics as reviewed by several groups (Stuart and Satija, 2019; Lee et al., 2020; Ma et al., 2020). These workflows aim to gather genomic, transcriptomic and proteomic information from each isolated cell and are expected to even be combined with other physiologic observations prior to these destructive analyses. We envision that similar developments are also on the horizon for the study of environmental microbial isolates, such as endophytic fungi.

### Genome Editing of Novel Fungal Isolates

Experimental verification of BGC identity in the native host is a major challenge in filamentous fungi. Several strategies for genome editing were recently reviewed in detail (Mei et al., 2019). Here, we'd like to emphasize only recent developments in the field of CRISPR/cas9 genome engineering of filamentous fungi that promise to also be applicable to organisms without an elaborate engineering toolbox at hand. As reviewed by Mei et al. (2019), Schuster and Kahmann (2019), Song et al. (2019), Ouedraogo and Tsang (2020), and Ullah et al. (2020), the CRISPR/cas9 strategy most commonly used in a variety of organisms including filamentous fungi relied mostly on the delivery of the Cas9 gene and guideDNA in expression cassettes for *in vivo* generation of the active cas9/guideRNA complex. This requires the availability of a suitable DNA shuttle, selective markers, and promoter and terminator sequences functional in the native host, which is often not available for new isolates. Recently, *in vitro* preparation of the Cas9-gRNA ribonucleoprotein complex and transformation into protoplasts of filamentous fungi has become available (Pohl et al., 2016; Al Abdallah et al., 2017; Wang et al., 2018). This system is reported to have a higher efficiency, fewer mistargets, and allows for rapid disruption using direct protoplast transformation. Although it has not been applied in endophytic fungi, this is a promising strategy to advance the bioprospecting effort of endophytic fungi with limited molecular tools. Not only in gene editing, CRISPR/Cas9 also advances the development of gene expression for biotechnological purposes using the recently developed CRISPR interference and CRISPR activation systems which offer novel strategies to regulate gene expression as reviewed by Zheng et al. (2019). Taken together, the use of CRISPR/Cas9 promises easier experimental verification of BGCs under investigation, and expedited construction of suitable expression systems for biotechnological processes involving endophytic fungi.

## Conclusion

Fungi are prolific producers of secondary metabolites and have been exploited for industrial production of pharmaceuticals and nutraceuticals for almost a century. Endophytic fungi, living in plant tissues are a very species-rich, yet somewhat elusive group of fungi that have been chased after by bioprospectors over the last three decades. Despite prevalent difficulties in cultivation and stability of metabolite production of these organisms, phenotypic studies describing the observation of novel metabolites are numerous. The real challenge appears to be the discovery of the underlying biosynthetic pathways that would allow the rational engineering, or refactoring of these pathways for industrial purposes. In our detailed literature survey presented in this review, we were only able to find nine examples of endophyte secondary metabolites, whose biosynthetic gene clusters were conclusively elucidated. The overall small number of whole genome sequences of endophytic fungi, and the often-low predictive value of known BGCs for fungal BGCs, make genome mining and BGC discovery challenging. We expect that some of these problems will be overcome with the availability of more functional genomics studies in fungi and especially endophytic fungi. For this endeavor, rule-independent approaches for BGC prediction will be instrumental, as well as advanced workflows for cultivation and experimental verification. Moreover, the increasing number of biochemical studies will allow for better training data sets for current and future machine learning-based platforms. Continuous improvements in the development of genetics and genomics tools have significantly supported the bioprospecting of natural products and will continue to do so.

## Author Contributions

RS and KH developed a concept for the review and collected the literature. RS wrote the manuscript with input from WQ and KH. All authors contributed to the article and approved the submitted version.

## Conflict of Interest

The authors declare that the research was conducted in the absence of any commercial or financial relationships that could be construed as a potential conflict of interest.

## Abbreviations

BGC: biosynthetic gene cluster
BLAST: basic local alignment search tool
CRISPR/cas9: clustered regularly interspaced short palindromic repeats/cas9
DHN: dihydroxynaphthalene
DNA: deoxyribonucleic acid
FACS: fluorescence-activated cell sorting
FAD: flavin adenine dinucleotide
FDA: United States Food and Drug Administration
FIND: Fungal one-step IsolatioN Device
GC: gas chromatography
HIV: human immunodeficiency virus
HPLC: high pressure liquid chromatography
IChip: isolation chip
JGI: Joint Genome Institute of the U.S. Department of Energy
LC: liquid chromatography
MS: mass spectrometry
NCBI: National Center for Biotechnology Information of the U.S. National Institutes of Health
ND: no data
NF-κB: nuclear factor kappa-light-chain-enhancer of activated B cells
NGS: Next Generation Sequencing
NMR: Nuclear Magnetic Resonance
NRPS: non-ribosomal peptide synthetase
OSMAC: one strain many compounds
PCR: polymerase chain reaction
PKS: polyketide synthase (hr—highly reducing, nr—non-reducing)
PK-DT: polyketide-diterpenoidqRT—quantitative PCR
RAL: resorcylic acid lactones
RMI: root microbiome interaction
RNA: ribonucleic acid
RNAseq: RNA sequencing technology
RT-qPCR: reverse transcriptase qPCR
U.S.: United States of America
REEIS: Research, Education and Economics Information System of the US Department of Agriculture
WGS: whole genome sequencing.
